# Supplementary material for: The burden of chronic diseases, disease-stratified exploration and gender-differentiated healthcare utilisation among patients in Bangladesh
Source: PLoS One. 2023 May 2;18(5):e0284117. doi: 10.1371/journal.pone.0284117 (PMC10153713; doi:10.1371/journal.pone.0284117)
Supplement: Attachment — Submitted filename: Accepted.docx [file pone.0284117.s002.docx]

Dear Prof. Baradaran

Editor of Plos-ONE

All comments were address by authors.

Regards
